# Disease management projects and the Chronic Care Model in action: baseline qualitative research

**DOI:** 10.1186/1472-6963-12-114

**Published:** 2012-05-11

**Authors:** Bethany Hipple Walters, Samantha A Adams, Anna P Nieboer, Roland Bal

**Affiliations:** 1 Institute of Health Policy and Management, Erasmus University, Rotterdam, The Netherlands

## Abstract

**Background:**

Disease management programs, especially those based on the Chronic Care Model (CCM), are increasingly common in the Netherlands. While disease management programs have been well-researched quantitatively and economically, less qualitative research has been done. The overall aim of the study is to explore how disease management programs are implemented within primary care settings in the Netherlands; this paper focuses on the early development and implementation stages of five disease management programs in the primary care setting, based on interviews with project leadership teams.

**Methods:**

Eleven semi-structured interviews were conducted at the five selected sites with sixteen professionals interviewed; all project directors and managers were interviewed. The interviews focused on each project’s chosen chronic illness (diabetes, eating disorders, COPD, multi-morbidity, CVRM) and project plan, barriers to development and implementation, the project leaders’ action and reactions, as well as their roles and responsibilities, and disease management strategies. Analysis was inductive and interpretive, based on the content of the interviews. After analysis, the results of this research on disease management programs and the Chronic Care Model are viewed from a traveling technology framework.

**Results:**

This analysis uncovered four themes that can be mapped to disease management and the Chronic Care Model: (1) changing the health care system, (2) patient-centered care, (3) technological systems and barriers, and (4) integrating projects into the larger system. Project leaders discussed the paths, both direct and indirect, for transforming the health care system to one that addresses chronic illness. Patient-centered care was highlighted as needed and a paradigm shift for many. Challenges with technological systems were pervasive. Project leaders managed the expenses of a traveling technology, including the social, financial, and administration involved.

**Conclusions:**

At the sites, project leaders served as travel guides, assisting and overseeing the programs as they traveled from the global plans to local actions. Project leaders, while hypothetically in control of the programs, in fact shared control of the traveling of the programs with patients, clinicians, and outside consultants. From this work, we can learn what roadblocks and expenses occur while a technology travels, from a project leader’s point of view.

## Background

The diagnosis of chronic disease has increased in the developed world in recent years, thought to be due to the aging population and the more successful treatment of acute illness. Many health care decision makers have been seeking methods of more efficiently and effectively treating chronic disease; frequently, these methods include the development and implementation of disease management programs [1]. While there are many specific definitions of disease management, for this article and for the projects discussed here, disease management has been defined as a “broad programmatic approach to chronic diseases, a comprehensive care chain that consists of diagnosis, treatment and support, as well as prevention, early detection, and self-management. The broad approach is set in multidisciplinary care standards and is organized around the patient and his illness, where possible tailored specifically to his environment” [2].

Disease management programs have been implemented in and tailored by health care providers around the world. As such, they can be understood as a ‘traveling technology’; traveling technologies are programs or health care principles that travel between a global (often academic) stage and the local practices and actions of health care leaders and providers. The traveling technologies also include the “translations that occur when an object travels from one place to another” [3]. In the process of traveling from the global (a general program or model) to the local (through application at a specific practice site), the programs become adapted and negotiated in their local contexts by project leaders, health care managers, clinicians, and patients. When viewed as a traveling technology, the local practices of health care providers and patients are as important as the global principles that frame those practices. Disease management programs are an excellent example of traveling technologies. While disease management programs originate from similar ideologies of patient-centered coordinated care, the local actions vary widely, as does the implementation and in some ways, the redefinition through the actions of local actors, of the tenets of disease management.

In this paper, we have collected data from the project directors and managers of five disease management programs in the Netherlands. The overall aim of the study is to explore how disease management programs are implemented within primary care settings in the Netherlands. For this paper, interviews done with project leadership teams conducted during the development and early implementation phases of the disease management programs have been analyzed from a traveling technologies viewpoint. This research provides a unique perspective on traveling technologies by showing how project leaders are crucial in the traveling of disease management programs at many levels, as the project leaders interpret, translate, adapt, and adopt the tenets of disease management programs to their local settings. This article uses the project leaders’ point of view to addresses the following question:

· How can disease management programs be understood as a traveling technology through the actions and reactions of project leaders?

### Disease management programs and the chronic care model

One of the more popular and pervasive models for framing disease management programs is the Chronic Care Model (CCM), which has influenced and been influenced by disease management programs. The Chronic Care Model “summarizes the basic elements for improving care in health systems at the community, organization, practice, and patient levels” [4]. The model, developed in the United States by a team led by Edward Wagner in the 1990s, is a synthesis of many components of disease management programs [5,6]. While there are other guides for disease management programs, none are as prolific as the Chronic Care Model; as such, the elements of the CCM will be used in this paper as a definition of the elements of disease management.

Much research has been or is currently being conducted on disease management programs, including those directly guided by the CCM [7,8], yet little research has focused on the implementation of disease management programs in their early phases. As well, there is need for more research now that disease management programs are increasingly being taken up in countries outside the US, as can be seen in Germany [9], New Zealand [10], the UK [11], and the Netherlands.

Disease management programs are increasingly popular in the Netherlands, due, in part, to changes within the health care financing and delivery system. Having undergone major reforms in 2006, the new regulated market health insurance system is continuing to adapt to the needs of insurers, health care providers, and patients [12]. Through the new system, every resident of the Netherlands is required to purchase health insurance. The cost of a basic health insurance plan, which covers preventative and essential curative services, is fixed; subsidies are available for those unable to pay. In 2008, the Ministry of Health, Welfare, and Sport emphasized the need to develop disease management programs for those with chronic diseases [13]. This resulted in the creation, in 2009, of a research and implementation program to improve upon the development and execution of disease management programs by the Netherlands Organization for Health Research and Development (ZonMw). ZonMw sent out a call for proposals for projects from practice groups in the Netherlands. The call for proposals for the projects emphasized the need for disease management programs that focus on self-management, co-morbidity, and/or information and communication technologies (ICT) in health care.

Twenty-two health care provider groups were granted funding for developing disease management programs. In exchange for funding, project leaders and clinicians agreed to develop and implement a disease management program that meets the needs of their patient population and to facilitate qualitative, quantitative, and economic research conducted at the site [14]; five were selected for further in-depth qualitative research, based on maximum variation between diseases and practices. Through the qualitative research, the research team will explore how disease management programs are implemented in the Netherlands by the project leaders and clinicians. This research also included how disease management programs move from plans to actions, how the programs are altered and tailored to the situation, and, toward the end of the project timeline, how clinicians and project leaders embed notions of disease management into every day practice. The clinicians at the project sites, under the guidance of their project leaders, selected the chronic condition that they would like to address to improve the health care of their patients. The project leaders developed project plans, often with intensive feedback from health care providers, and submitted the plans for funding to ZonMw. ZonMw funded the projects thought to be most likely to succeed and impact a large population of patients with chronic disease.

## Methods

The qualitative study is part of a larger effort to understand the impact of disease management programs on clinical systems, clinician behavior, patient perception of care, health outcomes, and on the financing of treatment. The larger goal of the qualitative component of the study is to understand the impact of developing and implementing disease management program on project leaders, clinicians, and patients; this paper looks at the impact on project leaders, while future work will look at the clinicians and patients. The study was approved by the Review Board of Erasmus MC.

### Settings/study sites

The five qualitative sites were selected as a purposeful sample on the basis of maximum variation, with respect to the planned development and use of information and communication technologies, interaction between primary and secondary care, type of intervention, role of the patient, and changes for professionals. They also represent a range of chronic conditions, including eating disorders, diabetes, and cardiovascular risk management.

### Research participants

Each study site has a project leader or a project leadership team, including project leaders who manage small projects within the large disease management project (known in this article as mini-project leaders), to guide the work of the program and project; many of these main project leaders or managers have had training prior to the study (as part of a Masters or as separate certification courses in health care organizations) in health care systems or disease management, though this was not a requirement of the programs or study. While many of the project leaders have education in health care management and program leadership, it is unclear to what degree project leaders were cognizant of the specific literature on the Chronic Care Model. The project leaders oversee the projects, work with clinicians at each GP office or department, collaborate with the research team on the economic, qualitative, and quantitative research, and develop and/or tailor the disease management program. Furthermore, the project leaders support projects by being contact points for ZonMw and for the two other supporting agencies, Vilans and Picasso for COPD. The project leaders are also expected to attend Inspiration Days, one-day conferences that address themes of interest to the projects, such as eHealth programs, program leadership, and patient education. The project leaders are granted much latitude in how the projects are conducted, though support and education is available on request.

### Design

At each of the five selected sites, semi-structured interviews were conducted with the project leaders and members of the project leadership team. Eleven interviews were conducted at the five selected sites with sixteen health care professionals; all of the interviews were conducted by the first author. The interviews were conducted at the program site in Dutch or English and ranged from 45 minutes to 90 minutes. The interviews were recorded; equipment malfunction resulted in two interviews with missing recordings. In all cases, in the event of equipment malfunction, the interviewer took detailed notes, including exact quotes from the interviewees, during the interviews. Interviews were transcribed verbatim. The interview quotes used in this manuscript were sent to the respondents in order to confirm use in the manuscript, as well as check any confusion in language. The respondents edited the quotes with minor changes and gave express permission to use the quotes in this manuscript.

Table 1 gives an overview of the sites; Table 2 summarizes the interviews conducted at each site. All project leaders were interviewed about their projects, their practice settings, and the changes being made as a result of the development and implementation of the disease management project. Table 3 outlines the major themes uncovered in the data collected from the interviews, including the themes covered in this manuscript, as well as other themes that will be explored further in future articles.

**Table 1:** Projects selected and interviewed for qualitative research

**Project site**	**Chronic illness**	**Team**	**Program**
Clinic for eating disorders and addictions	Eating disorders	On-site leadership team 1 project leader (MD) 1 project manager (Social worker) 4 mini-project leaders	4 fold project to reduce barriers to access to support and treatment for eating disorders
GP practice group	Cardiovascular risk (CVR) management	On-site leadership team 2 project leaders (MDs) 1 project manager (RN)	Manage CVR through patient portals, patient care plans, and interdisciplinary care teams
GP practice group	Diabetes	Consultant leader 1 project leader (Health care consultant)	Train clinicians and other health care givers to manage diabetes in first line care
GP practice group	Chronic obstructive pulmonary disease (COPD)	Consultant leader 1 project leader (Health care consultant)	Find and recommend an effective ICT system and an expert COPD training team visiting each practice
GP practice group	Multi-morbidity	Consultant leaders 2 project leaders (MD, RN)	Develop integrated approaches for the management of co-morbidity and chronic illness

**Table 2:** Interviews conducted

**Project site**	**Interviews**
Clinic for eating disorders and addictions	One-on-one interview with project leader
	One-on-one interview with project manager
	Three one-on-one interviews with mini-project leaders
	Joint interview with mini-project leader and
	department head
	Total interviews:
	6
GP practice group	Joint interview with project leaders
	One-on-one interview with project manager
	Total interviews:
	2
GP practice group	One-on-one interview with project leader
	Total interviews:
	1
GP practice group	Joint interview with project leader and outside expert
	Total interviews:
	1
GP practice group	Joint interview with project leaders
	Total interviews:
	1

**Table 3:** Themes that emerged from the qualitative research

**Chronic care model**	
Integration	
Time	
Roles	
Disease management	
Diabetes	
Eating disorders	
CVRM	
Multi-morbidity	
ICT/Electronic Health Record	
Internet	
Stepped care	
Ketenzorg	
Reducing the use of specialists	
Barriers	
Communication	

Baseline interview questions were developed from the literature in disease management [15,16], the Chronic Care Model [17-19], and conditions related to each site [20-24]. Further questions were developed after a review of the data collection methods of the quantitative and economic aspects of the study; the first author also met regularly with the economic and quantitative researchers and discussed the qualitative data collection to ensure harmony and inclusiveness of the qualitative data collection within the overall study goals. The qualitative data collection questions also cover areas that are not fully addressed by economic and quantitative aspects of the study to ensure complete data capture for the entire study, such as barriers to implementation at this point, challenges to the development of the project, an exploration of the team dynamics, communication strategies used with the project team, and any (un)pleasant surprises that they have encountered. Project leaders were asked about their own experiences in developing, tailoring, and implementing the disease management programs, as well as any interest or criticisms that they had heard from other professionals and clinicians involved in the disease management programs. While the answers to these questions were analyzed from a traveling technology framework, the questions themselves map back to the primary aim of the study, understanding how disease management programs are implemented in primary care in the Netherlands; Table 4 has a sample of the interview guide. From this information, the research team was able to gather information about how the disease management programs were being implemented in the sites, as well as how the disease management programs were best understood as traveling technologies as they were implemented and altered by the project leaders.

**Table 4:** Sample from the interview guide

	1 Why were the projects developed?	
	2 What is the need?	
	3 What are the barriers to implementation?	
	4 How does communication happen?	
	a As a team	
	5 What has feedback been to the implementation from staff?	
	6 How has your team helped in the creation and implementation of the project?	
	7 How was your team developed?	
	8 What does it mean to have a disease management system or project?	
	9What good surprises have you had?	

### Analytical approach

Analysis was inductive and interpretive, based on the content of the interviews; in line with the inductive analytic tradition, project leaders were frequently asked to define terms that are otherwise taken for granted as a shared understanding, such as ‘disease management’ [see Table 4. The content of the interviews focused on the implementation of the disease management programs by the project leadership team, communication and team dynamics between members of the project leadership team, the clinicians, and the patients, and changes in health care practices, based on the diseases addressed in the programs. This method of inductive analysis allows for the development of general themes mapped back to the literature [25]. As disease management programs contain many of the aspects of a traveling technology (traveling expenditures, movement from global to local arenas), the data collected through interviews with the project leaders was most clearly understood through a traveling technologies framework. All research questions have been analyzed using the same method. The data was coded by the first author and verified through close reading of the quotes in their original language and their translation, when appropriate, by the second author, SA.

## Results

From the empirical data, four disease management themes, as defined by the Chronic Care Model, emerged:

(1) changing the health care system,

(2) patient-centered care,

(3) technological systems and barriers, and

(4) integrating projects into the larger health care system.

Illustrative quotes from these themes have been selected from this article and are seen below in italics and off-set from the text. Through defining disease management through these Chronic Care Model themes, the research team was able to understand and explore how disease management programs travel. This research shows how project leaders manage and interact with the members of the disease management programs, and ultimately, the patients and clients impacted by the disease management programs, as well as how project leaders view the travel expenditures associated with the programs.

### Changing the health care system

Project leaders and project leadership teams, including project managers and mini-project leaders, frequently tied their disease management project work to larger efforts in health care and in their health care system’s delivery of care in specific. Project leaders sited the goals of the programs as the impetus to plan (and in the future, put in place) changes, as well as the goal of improving patient care.

*The end goal is to reach more people in a better way with a continuous quality of care during the, on average, 5 to 7 year illness period. That’s the idea. That’s ambitious. Yeah, it’s very exciting because we have to change the whole way of thinking here and change the whole organizational model as well… So we have inner barriers to continuity in care as well. Lack of flexibility there as well, so we need to reorganize internally this year*. (Interview with E, project director for the eating disorder project)

The latitude afforded to the project leaders in developing their programs means that the translation from the project plan on paper to action in practice can be done in a number of ways: as a drastic reorganization, in phases, or first as a project with later spoken or unspoken plans for integration into the system. It also involves translation work in relationship to stakeholders:

*I have to tell [the GPs] that it’s just a project and that we will need an evaluation of the project before it comes to be common practice*. (Interview with X, project leader with the CVR project)

### Patient-centered care

Patient-centered care, as defined by the Chronic Care Model through the Self-Management Support element of the model, highlights “the patient’s central role in managing their health” [26]. While the majority of the project leaders did not mention the Chronic Care Model as inspiration for the changes that they plan to make, a few did and directly tied their actions to the model.

*For example, that together with the patient, you look at the self-management of that patient and that you look according to the Chronic Care Model, like yeah, I have to work along with the patient on all the factors and not only give information, because that isn’t going to help that patient. That’s what is important, that I can see now in a broad perspective and then you can make the most of giving care*. (Interview 1 with H, project manager with the CVR project)

*And then another thing was the Chronic Care Model is a patient-centered model. And we are not used to working [in a] patient-centered [way]. It’s becoming more and more [popular]. What’s new in our system is that we choose to work in a patient-centered way.* (Interview with X, project leader with the CVR project)

However, as noted in the quote above, working in a patient-centered way is a change within the system, a change that costs time and effort. This expenditure of time and effort is for a common goal: involving patients in their health care to improve their health.

*Well and involving the patient now is one of the most important things because what we’ve discovered and what worldwide they discovered is that lifestyle change is one of the most important issues that can make a difference on the long term, but is also one of the most difficult things to realize. And the only way to do that is getting patients involved, and getting them to participate in their own disease*. (Interview with B, project leader for the diabetes project)

Project leaders emphasize how becoming more patient-centered impacts the providers and how providers will need to change how care is delivered.

*The two things that are the most important are the self management of the patients themselves because the patient is the main issue of gaining a long term benefit out of the system. So that’s one thing. But what’s also necessary is that the care givers, the doctors, the physicians, the nurses, they have to make a switch in not only being a health care giver but being a coach, being able to give the support to the patient that they can make their own self-management system and that they can make their own choices and that will really make a difference, instead of the choice of the health care giver.* (Interview with B, project leader for the diabetes project)

Other project leaders place emphasis on the changed role of the patient and their involvement in their care process.

*But it’s the process of the client, not the process of the one who’s giving the support. So the process of the client is leading. That’s a difficult part of it, but the essential part of it. Do you know what I mean? I mean… I can tell you what you’ve got to do to recovery, what’s good for you. But that won’t be necessarily your way. …I’ve got to connect with your approaches. And look together with you and support you to empower you to find your own path in recovery.* (Interview with P, mini-project leader for the eating disorder project)

Patient-centered care can be a challenge for health care providers, as it may require them to change the ways in which care is presented and delivered to patients. Health care providers must think and act in new ways, as well as continue to alter their health care systems to sustain this new way of providing care.

*The challenge is to know who needs support and who can self- manage.* (Interview with H, project manager for the CVR project)

*To learn to think about the client is the most difficult thing [to do]. To really think about the client. Not that I know what is best for the client. The client knows best. That’s a hell of a job.* (Interview with K, project manager for the eating disorder project)

### Technological systems and barriers

For the project leaders, implementing a disease management program is a process of harmonizing the movement of information and actions between practice sites, patients, and the project leaders. In all cases, this change to the health care system involves new communication technology. The improvement or addition of a technological system is a common way to change the mode of health care delivery and is emphasized in the call for proposals for the projects. The planned changes in the projects include the development of patient portals to assist patients in self-management, patient health records, computerized communication systems to connect general practitioners and other health care professionals, and websites for patients/clients to connect with clinicians and others. These changes are seen as important for organizing and improving care for both patients and clinicians.

*So now all general practices have their own registration and programs. And we think it’s very important that the general practices and the hospital work together and can see the registration and can communicate together. And we think that we need another program for that.* (Interview 1 with AC, project leader for the COPD project)

*But what is especially important and that’s where we are now spending a lot of time is to properly organize care with the GPs who work on a computer.* (Interview with H, project manager for the CVR project)

However, ICT systems are also seen as a barrier, in that the development and implementation of the systems is costly in both time and effort. Project leaders must also spend time prioritizing and in some ways, limiting, the focus of the ICT system.

We have a lot of barriers. First barrier is with the ICT system. It’s not already finished… It was a long step to come so far as we are now. (Interview 1 with X, project leader with the CVR project)

*The fact that the software builder couldn’t deliver what they said they would deliver [was a barrier]. And still now we do not really have the perfect system and the perfect system does not exist, I know. But there are too many things that are really what we want.* (Interview with B, project leader for the diabetes project)

*And you have the program, and it’s sort of the same, and it works on the mobile phone. And E. thought that it would be a program that worked on the Internet and on the mobile phone. And there are two programs, but the content is different, so you have only prevention on the Internet or after care on the SMS. So there were all kind of technology things that had to be discussed before we decided what to do.* (Interview with M, mini-project leader for the eating disorder project)

Patients and clients, too, play a role in the development of an ICT system. Depending on the system, patients and clients will be able to interact directly with clinicians through a website or patient portal, clinicians at multiple locations will have the ability to interface with a patient’s record, and/or project leaders and clinicians will be able to review the information of large sets of patients for patterns and quality control.

*They [the patients] can choose their treatments. And all those steps we have laid out in our ICT scheme.*(Interview with X, project leader with the CVR project)

However, not all patients are expected to want to directly participate in an ICT system or have the skills to do so at this time.

*Well there are patients who say “I do not want that my information is put in the software system” so we have a form that they can sign if they do not what that.* (Interview with B, project leader with the diabetes project)

### Integrating projects into the larger health care system

As the projects are funded for a short amount of time, the project leaders recognize the need to integrate the projects into the larger health care system if they want to make lasting changes to the delivery of health care. This effort to integrate projects into routine care often involves the development of program plans, budgets, the hiring of new staff, and/or the training of existing staff. Some project leaders see the projects as an opportunity to expand the scope of the projects and create a system for the management of chronic disease in general at their sites.

*The third step is to make the disease management program a multi-morbidity program. These steps are further integration into chronic care. The project is a model for all future chronic care programs. We don’t believe in a system for separate projects formulti-morbidity… One program.* (Interview with V, project leader for the multi-morbidity project)

*And the second thing there is trying to make it more a disease management than only one chronic care model. For COPD and cardiovascular, we know that we are going to introduce also the chain system, so we’re trying to make the base ready for other chronic care systems.* (Interview with B, project leader for the diabetes project)

*Other members of the project leadership team are working with clinicians to imbed the current changes into the current health care delivery system. So they have to integrate it in their daily work…in their practice. That’s a very big step to get it implemented there and instruct all the other employees in the practices.* (Interview with R, outside expert at the COPD project)

However, project leaders find that working with clinicians to think beyond the project and beyond their currently defined roles can be a challenge.

*That’s the biggest challenge. Because we are all professionals at this moment. It’s very difficult to connect the clinic with the outpatient clinic. Ah, I think that we are, as professionals, able to want to look further than our little business. It’s the same old story with all the professionals in health care.* (Interview with K, project manager for the eating disorder project)

For the project leaders, the disease management programs are an iterative process, with ongoing efforts made to improve the programs and the care that they help provide.

*The program is not the answer, only an answer. We have to have the courage to change again without always being on the move.* (Interview with S, department head at the eating disorders project)

## Discussion

In this study, disease management programs, as defined by the elements of the Chronic Care Model, are analyzed as a traveling technology. A traveling technology refers to the translations, adaptations, and expenditures that occur when an object or program moves from one location to another; traveling is more than the translation of the disease management projects, as it encompasses the translation of the disease management programs to the local setting, but focuses on the travel expenditures and travel documents created in the process [27]. As a result, project leaders play an important role in this process, especially during the development and early implementation phases. It’s important to note that the traveling expenditures of the programs are much more than financial and include the social costs and changed expectations, the administrative effort, and the altered obligations for patients and staff; these traveling expenditures are often hidden and in many ways, unexpected by the project leaders.

Through management and organizational work, the project directors serve as ‘travel guides’ for the programs, as they oversee the expenditures of the programs, help guide the travel of the disease management programs to an individual clinician’s offices, assist clinicians involved in the projects in the travel of the programs to and with patients, and connect the disease management programs to a global disease management community. Much of this work involves the creation and management of documents involved in each of the aspects of the disease management programs; these documents include framing documents, such as revised project plans, and communication documents, such as emails to clinicians and newsletters. Each aspect or theme of disease management (changing the health care system, patient-centered care, technological systems and barriers, and integrating projects into the larger health care system) involves the creation or management of documents or communications (including telephone and in-person communication) by the project leaders. As can be seen in the interviews, project leaders were frequently communicating with the clinicians and editing ICT plans to match the available computer programs. These documents travel the technology, both literally (as a newsletter moves from the project director to the clinician) and figuratively (as the documents house strategies and networks for the traveling of the programs).

While disease management programs have been widely touted as a method of reducing costs in health care delivery, research shows that the implementation costs can have a large financial impact [28]. However, the costs of developing and implementing a disease management program are more than money and effort. These traveling expenditures include the making and managing of program documents, the developing and maintaining of relationships and networks, the re-shaping of roles and responsibilities of patients and clinicians, and the adapting and moving of regulations and policies [29]. In the five disease management programs, we see that project directors are aware of these traveling expenditures. This can be seen in the project leader’s statements about the development and implementation of ICT systems, as well as in discussions of clinician training. The time needed to refine and develop the programs, to manage the timeline of the programs in relation to the ICT system, and to negotiate relationships with software developers are all expenditures that the project director must manage, even when many of the expenditures are out of his/her control.

While it might be assumed that as ‘travel guides’, the project directors are in control of the projects, this is not unvaryingly true. In fact, control and traveling of the programs shifts between the project director, outside contractors, clinicians, and patients. In some cases, the project director actively moves the control, such as by working to change the role of the patient to one that is more self-managed, while in other cases, both the control of the programs and its traveling moves in spite of the actions and desires of the project director. This movement of control can be seen in the project directors’ statements about the development of ICT programs and in their thoughts on the challenges associated with the changed roles for clinicians in regard to patient self-management. However, while the project director may be (in title and in role) in charge of the project, the traveling of the disease management programs is also reliant on the actions (and inactions) of the local clinicians, patients, and outside contractors.

The disease management programs, in line with the elements of the Chronic Care Model, are implementing some form of computer-based health system. These computer-based health systems are designed to enable the flow of information between clinicians in multiple locations, connect clinicians and patients, and organize the work of the programs through communication and the posting of project plans and meeting notes. Yet computer-based health systems in general are still a work in progress for the technological developers and for the end-users [30]. Implementing computer-based systems is a major undertaking for health care organizations, needing support, organizational, cultural, and technical changes [31]; even when implemented, the computer-based system may not provide the improvement in care desired, but may increase mistakes in medical record documentation, medication dosing, and may, in fact, be more difficult for clinicians to use [32]. Developing and using computer-based systems to travel the program to and between health care providers, project leaders, and patients is not a simple task but is a ‘mutual shaping’ of expectations and goals [33]. This mutual shaping can be seen in revised project plans for ICT systems, as well as extended timelines.

Better care for patients with a chronic condition is one of the main goals of the disease management programs. Project directors see disease management as a patient-led journey (*it’s the process of the client)* that focuses on the needs of the patient (*the client knows best*) through self-management. Project directors work to travel, to move, both the patients to the program (*to move clients to the clinic)* and the program to the patients. In line with the traveling technology framework, project directors use movement verbs when discussing patient involvement in the disease management program, highlighting the actions that the project directors are taking to move the program to the patient. Yet, in line with literature on self-management [34], project directors see that self-management has challenges and limitations (*the challenge is to know who needs support and who can self-manage*). Through their understanding of these challenges and limitations, project directors work to find ways to travel the programs to the patient in a way that is appropriate and thought to be acceptable to the patients (*one is to provide adequate information but not in professional language but in language that appeals to teenagers, so anecdotally, but to provide information which is at least scientifically accurate).*

While traveling the programs to patients and to the clinicians involved in the programs, project directors also see their work as connected to a larger global arena. Globalized language is apparent in the projects (*all the professionals in health care, what worldwide they discovered*), tying the project leaders and the projects to a larger movement in health care. The project travels from the global, as defined by the elements of the Chronic Care Model, to the local health care providers. Project leaders are aware of the influence of outside models, with some referencing the chronic care or the Chronic Care Model as an influencing factor, and are cognizant of the longer term implications of the projects (*The project is a model for all future chronic care programs*.). This awareness leads to broader efforts when developing the programs, as well as a willingness to be influenced by larger trends within health care (*look according to the Chronic Care Model)*. The project leaders are responsible for traveling to and from the large global sphere.

Research on disease management programs and the Chronic Care Model is increasingly relevant, as health care systems are turning to disease management programs to treat the rising number of patients with chronic diseases. While other research has focused on the implementation of the Chronic Care Model in Belgium [35], where the care of patients with chronic disease is delivered in primary care setting within a limited structure, this research focuses on the primary care setting in the Netherlands; in the Netherlands, the primary care setting is the typical setting of disease management programs for the care of patients with chronic diseases, including those studied in this article. Similar to the findings in this article (*I have to tell [the GPs] that it’s just a project)*, research conducted in one large health care organization implementing programs based on the Chronic Care Model found that physician engagement could be difficult due to lack of commitment, lack of time, and change fatigue [36]. Other researchers in the US have shown that while health care organizations using collaborative teams can make substantial changes in the delivery of care for patients with chronic diseases, these changes can be difficult to maintain at the same level of intensity [37]. As the research conducted in this article is during the planning and early implementation stages of disease management programs, it will be useful to observe the evolution of the disease management programs as a traveling technology over time.

This article presents information that could benefit project leaders of disease management in understanding the longer term implications of their work in both global and local arenas. This information could be especially useful to project leaders within the Netherlands or who are new to disease management programs. This data shows how project leaders adapt to and adopt new systems. The qualitative data on which this article builds give important insights into how project leaders, especially project leaders who are new to disease management programs, struggle with and overcome challenges involved in interacting with a traveling technology such as the Chronic Care Model. Understanding how project leaders struggle with and overcome these challenges will help facilitate the development of better supportive structures for disease management projects, as well as the development of more comprehensive project plans and budgets.

However, the paper is limited by the number of interviews. As the interviews were conducted in the early stages of the programs, only 11 interviews are available. In the case of the eating disorders project, where multiple mini-projects are being conducted as part of the larger disease management project, each mini-project leader was interviewed. As this research follows the project leaders and programs over time, this will be improved in future papers and will show not only how project leaders work with and through the Chronic Care Model, but will also reveal how GPs and other clinicians adapt and adopt to the project leader’s guidance. While the limited number of interviews may impact the conclusions, the use of these interviews is still relevant; the mini-project leaders serve a similar role as the project leaders at other sites, overseeing the projects, the project staff, the timeline, communication with patients and/or other clinicians, and the content of the mini-project.

## Conclusion

This study has revealed that disease management project leaders serve as ‘travel guides’ when a disease management program is a traveling technology and adds to the understanding of how disease management programs are developed and implemented in the primary care setting. Through the project leaders’ interpretation, translation, adaption, and adoption of the tenets of disease management, as well as through the management of travel expenditures, the disease management programs travel not only from global notions of disease management, but more importantly, the programs travel within the network of the project teams: from the project plan to implementation, from the project leader to the clinicians, from the clinicians to the patients.

As ‘travel guides’, project leaders should be aware that the impact of their work is both deeper and broader than they may realize, as the disease management programs are traveling throughout their project teams. In many ways, project leaders set the overall tone of the project – the focus and the sentiments behind the disease management program. Their work not only guides the overall project, but impacts the interaction of one clinician with one patient, as well as traveling to the broader disease management arena through participation in research, through the development of care consortiums, and through the honing of standards and protocols within the Dutch health care system.

Understanding the beginning stages of implementing a disease management program (at the project leader level, as well as at the health systems level) can be helpful for other programs and health systems. Other health care clinicians can benefit from this work by understanding that the development and implementation of disease management programs is also the implementation and development of a traveling technology, with associated traveling expenditures and roadblocks. Through this, other project leaders can understand their work has broader implications both within their own program and as part of a larger disease management community. Much can be done at the project development phase to aim for smoother implementation, though every project will still face travel expenditures. Project leaders should pay more attention to the lasting effects of their work in all of the arenas it touches. This attention can come through more forethought on the implications of their project as a traveling technology, more research on the various elements involved in the project, such as ICT systems, and more funding allocated to project leadership and project leadership development during the development and early implementation phases of the project. Project plans can allow for more hours for project leadership, as well as a more flexible timeline to allow for overcoming roadblocks and overseeing traveling expenditures.

## Competing interests

The authors declare that they have no competing interests.

## Authors’ contributions

BHW conducted the interviews. BHW, SA, and RB analyzed the quotes. All authors contributed to the introduction, discussion, and the use of the literature in the paper. All authors have read and approved the final manuscript.

## Pre-publication history

The pre-publication history for this paper can be accessed here:

http://www.biomedcentral.com/1472-6963/12/114/prepub

## References

[B1] Ellrodt G Cook DJ Lee J Cho M Hunt D Weingarten S Evidence-based disease management JAMA 1997 278 20 1687 1692 10.1001/jama.1997.03550200063033 9388089

[B2] Ministerie van Volksgezondheid, Welzijn en Sport Programmavoorstel diseasemanagement chronische ziekten 30 November 2007

[B3] Nielsen AJ Traveling technologies and transformations in health care. PhD thesis 2010 Copenhagen Business School

[B4] Improving Chronic Illness Care About ICIC and Our Work http://www.improvingchroniccare.org/index.php?p=About_US&s=6 Accessed on 2/2/2011

[B5] Wagner EH Chronic disease management: what will it take to improve care for chronic illness? Effective Clin Pract 1998 1 1 2 4 10345255

[B6] Bodenheimer T Wagner EH Grumbach K Improving primary care for patients with chronic illness JAMA 2002 288 15 1909 1914 10.1001/jama.288.15.1909 12377092

[B7] Glasgow RE Orleans CT Wagner EH Does the chronic care model serve also as a template for improving prevention? Milbank Q 2001 79 4 579 612 iv-v 10.1111/1468-0009.00222 11789118 PMC2751207

[B8] Coleman K Austin BT Brach C Wagner EH Evidence on the Chronic Care Model in the new millennium Health Aff 2009 28 1 75 85 10.1377/hlthaff.28.1.75 PMC5091929 19124857

[B9] Szecsenyi J Rosemann T Joos S Peters-Klimm F Miksch A German diabetes disease management programs are appropriate for restructuring care according to the chronic care model Diabetes Care 2008 31 1150 1154 10.2337/dc07-2104 18299443

[B10] Rea H Kenealy T Wellingham J Moffitt A Sinclair G McAuley S Goodman S Arcus K Chronic care management evolves towards integrated care in counties Manukau. New Zealand J New Zealand Med Assoc 2007 120 1252 17460739

[B11] Lewis R Dixon J Rethinking management of chronic diseases BMJ 328 220 10.1136/bmj.328.7433.220 PMC318497 14739194

[B12] Schäfer W Kroneman M Boerma W van den Berg M Westert G Devillé W van Ginneken E The Netherlands: health system review Health Syst Transit 2010 12 1 1 229 21132996

[B13] Schäfer W Kroneman M Boerma W van den Berg M Westert G Devillé W van Ginneken E The Netherlands: health system review Health Syst Transit 2010 12 1 1 229 21132996

[B14] Lemmens KM Rutten-Van Molken MP Cramm JM Huijsman R Bal R Nieboer AP Evaluation of a large scale implementation of disease management programmes in various Dutch regions: a study protocol BMC Health Serv Res 2011 11 1 6 10.1186/1472-6963-11-6 21219620 PMC3025828

[B15] Ellrodt G Cook DJ Lee J Cho M Hunt D Weingarten S Evidence- based disease management JAMA 1997 278 20 1687 1692 10.1001/jama.1997.03550200063033 9388089

[B16] Weingarten SR Henning JM Badamgarav E Knight K Hasselblad V Gano A Ofman JJ Interventions used in disease management programmes for patients with chronic illness—which ones work? Meta­analysis of published reports BMJ 2002 325 7370 925 10.1136/bmj.325.7370.925 12399340 PMC130055

[B17] Wagner EH Austin BT Davis C Hindmarsh M Schaefer J Bonomi A Improving chronic illness care: translating evidence into action Health Aff 2001 20 6 64 78 10.1377/hlthaff.20.6.64 11816692

[B18] Oprea L Braunack-Mayer A Rogers WA Stocks N An ethical justification for the chronic care model Health Expect 2010 13 1 55 64 10.1111/j.1369-7625.2009.00581.x 19906213 PMC5060517

[B19] Pearson ML Wu S Schaefer J Bonomi AE Shortell SM Mendel PJ Marsteller JA Louis TA Rosen M Keeler EB Assessing the implementation of the chronic care model in quality improvement collaboratives Health Serv Res 2005 40 4 978 996 10.1111/j.1475-6773.2005.00397.x 16033488 PMC1361183

[B20] de la Rie SM Noordenbos G van Furth EM Quality of life and eating disorders Qual Life Res 2005 14 1511 1522 10.1007/s11136-005-0585-0 16110931

[B21] Adams S Smith PK Allan PF Anzueto A Pugh JA Cornell JE Systematic review of the chronic care model in chronic obstructive pulmonary disease prevention and management Arch Intern Med 2007 167 6 551 561 10.1001/archinte.167.6.551 17389286

[B22] Norris SL Nichols PJ Caspersen CJ Glasgow RE Engelgau MM Jack L Isham G Snyder SR Carande-Kulis VG Garfield S Briss P McCulloch D The effectiveness of disease and case management for people with diabetes Am J Prev Med 2002 22 4 Suppl 15 38 11985933 10.1016/s0749-3797(02)00423-3

[B23] Peters-Klimm F Olbort R Campbell S Mahler C Miksch A Baldauf A Szecsenyi J Physicians’ view of primary care-based case management for patients with heart failure: a qualitative study Int J Qual Health Care 2009 21 5 363 371 10.1093/intqhc/mzp032 19684033 PMC2742393

[B24] Moser A van der Bruggen H Widdershoven G Spreeuwenberg C Self-management of type 2 diabetes mellitus: a qualitative investigation from the perspective of participants in a nurse-led, shared-care programme in the Netherlands BMC Publ Health 2008 18 8 91 10.1186/1471-2458-8-91 PMC2292711 18366665

[B25] Creswell JW Research Design: qualitative, quantitative and mixed method approaches 2003 Thousand Oaks CA: Sage Publications

[B26] Improving Chronic Illness Care The Chronic Care Model Self Management Support http://www.improvingchroniccare.org/index.php?p=Self- Management_Support&s=22 Accessed on 2/2/2011

[B27] Nielsen AJ Traveling technologies and transformations in health care. PhD thesis 2010 Copenhagen Business School,

[B28] Gandjour A A model to predict the cost-effectiveness of disease management programs Health Economics 19 6 697 715 19582700 10.1002/hec.1503

[B29] Nielsen AJ Traveling technologies and transformations in health care PhD thesis 2010 Copenhagen Business School

[B30] Wears RL Berg M Computer technology and clinical work: still waiting for godot JAMA 2005 293 10 1261 1263 10.1001/jama.293.10.1261 15755949

[B31] Pirnejad H Bal R Berg M Building an inter-organizational communication network and challenges for preserving interoperability Int J Med Inform 2008 77 12 818 827 10.1016/j.ijmedinf.2008.05.001 18579436

[B32] Ash JS Berg M Coiera E Some unintended consequences of information technology in health care: the nature of patient care information system-related errors J Am Med Inform Assoc 2004 11 2 104 112 14633936 10.1197/jamia.M1471 PMC353015

[B33] Aarts J Doorewaard H Berg M Understanding implementation: the case of a computerized physician order entry system in a large Dutch university medical center J Am Med Inform Assoc 2004 11 3 207 216 10.1197/jamia.M1372 14764612 PMC400519

[B34] Rogers A Kennedy A Nelson E Robinson A Uncovering the limits of patient-centeredness: implementing a self-management trial for chronic illness Qual Health Res 2005 15 224 239 10.1177/1049732304272048 15611205

[B35] Sunaert P Bastiaens H Feyen L Snauwaert B Nobels F Wens J Vermeire E Van Royen P De Maeseneer J De Sutter A Willems S Implementation of a program for type 2 diabetes based on the Chronic Care Model in a hospital-centered health care system: “the Belgian experience” BMC Heal Serv Res 2009 9 152 10.1186/1472-6963-9-152 PMC2757022 19698185

[B36] Hroscikoski MC Solberg LI Sperl-Hillen JM Harper PG McGrail MP Crabtree BF Challenges of change: a qualitative study of chronic care model implementation Ann Fam Med 2006 4 4 317 326 10.1370/afm.570 16868235 PMC1522157

[B37] Pearson ML Wu S Schaefer J Bonomi AE Shortell SM Mendel PJ Assessing the implementation of the chronic care model in quality improvement collaboratives Heal Serv Res 2005 40 4 978 996 10.1111/j.1475-6773.2005.00397.x PMC1361183 16033488

